# miR-26a Potentially Contributes to the Regulation of Fatty Acid and Sterol Metabolism In Vitro Human HepG2 Cell Model of Nonalcoholic Fatty Liver Disease

**DOI:** 10.1155/2018/8515343

**Published:** 2018-09-30

**Authors:** Omaima Ali, Hebatallah A. Darwish, Kamal M. Eldeib, Samy A. Abdel Azim

**Affiliations:** ^1^Medicinal Chemistry and Molecular Pharmacology (MCMP), College of Pharmacy, Purdue University, West Lafayette, IN 47907, USA; ^2^National Organization of Drug Control and Research (NODCAR), Cairo 12553, Egypt; ^3^Department of Biochemistry, Faculty of Pharmacy, Cairo University, Cairo 11562, Egypt; ^4^Faculty of Pharmaceutical Sciences and Pharmaceutical Industries, Department of Pharmacology, Toxicology and Biochemistry, Future University, Cairo, Egypt

## Abstract

Nonalcoholic fatty liver disease (NAFLD) is a metabolic-related disorder ranging from steatosis to steatohepatitis, which may progress to cirrhosis and hepatocellular carcinoma (HCC). This study aimed at assessing the regulatory and protective role of miR-26a on lipid metabolism and progression of NAFLD in human HepG2 cells loaded with free fatty acids (FFA). Lentivirus expressing miR-26a or negative control miR was used to transduce HepG2 cells and to establish stable cell lines. Gain or loss of function using an miR-26a inhibitor was used to compare triglyceride content (TG), total cholesterol level (CL), total antioxidant capacity (TAC), malondialdehyde (MDA) and the level of apoptosis. In addition, quantitative reverse transcription polymerase chain reaction (qPCR) was used to assess the mRNA levels of lipogenesis, TG synthesis, storage genes, inflammatory and fibrogenic markers, and autophagic besides endoplasmic reticulum (ER) stress markers after gaining or losing the function of miR-26a. miR-26a levels decreased in response to FFA in human HepG2 cells. After the establishment of a stable cell line, the upregulation of miR-26a resulted in the downregulation of TG, CL, and MDA levels, through regulating mRNA levels of genes involved in lipid homeostasis, ER stress marker, inflammatory and fibrogenic markers. Nevertheless, there was a marked increment in the mRNA expression of autophagic marker genes. Moreover, miR-26a overexpression protects the cells from apoptosis, whereas inhibition of miR-26a, using an anti-miR-26a oligonucleotide, decreased the expression of miR-26a which potentially contributes to altered lipid metabolism in HepG2 cells loaded with FFA. In conclusion, these findings suggested that miR-26a has a crucial role in regulating fatty acid and cholesterol homeostasis in HepG2 cells, along with the offered protection against the progression of NAFLD *in vitro*. Hence, miRNAs could receive growing attention as useful noninvasive diagnostic markers to follow the progression of NAFLD and to identify novel therapeutic targets.

## 1. Introduction

Nonalcoholic fatty liver disease (NAFLD) represents the most common form of chronic liver disorder worldwide. It is characterized by hepatic steatosis, without significant alcohol consumption, which represents the hepatic manifestation of metabolic syndrome [1]. This abnormal hepatic disorder is due to excessive de novo lipogenesis, reduced *β*-oxidation, and/or reduced lipid excretion [2]. Simple steatosis can progress to nonalcoholic steatohepatitis (NASH), leading to a more severe stage that involves fibrosis, cirrhosis, hepatocellular carcinoma (HCC), and liver failure [3].

Pathogenesis of NAFLD is a multistep procedure that is not entirely delineated. Diverse speculations have been explained, driving at first to the two-hit theory. Hepatic lipid accumulation secondary to a high-fat diet, obesity and insulin resistance, acts as the first hit, which sensitizes the liver to further insults acting as a “second hit” that induces inflammation and fibrogenesis [4]. Notably, the second hit can be an assortment of variables including reactive oxygen spices (ROS) and endoplasmic reticulum (ER) stress [5]. With regard to increased free fatty acid (FFA) supply to hepatocytes, oxidative stress can be produced due to ROS and lipid peroxidation generated during the metabolism of fatty acids [6].

This initial “two-hit” theory for explaining the progression from NAFLD to NASH is now being replaced by multiple hits [7]. Furthermore, gut microbiota [8] and genetic polymorphism such as PNPLA3 and TM6SF2 [9] are also implicated in the development and progression of the disease.

MicroRNAs (miRNAs) are a class of endogenously expressed small noncoding RNAs (19–22 nucleotides) that regulate gene expression at the posttranscriptional level by binding to the 3′-untranslated region (UTR) of target genes [10]. They repress gene expression through two major mechanisms: inhibiting translation or targeting gene degradation [11]. miRNAs have recently received great attention because they are usually dysregulated in a variety of diseases; for instance, miR-125 upregulation by estrogen was documented to protect female mice from NAFLD [12]. Another research has also revealed that miR-21 reduced the levels of triglyceride along with cholesterol by targeting HMGCR [13]. Furthermore, another study observed an alteration in key miRNA processing component (Dicer1, Drosha, and DGCR8), together with other seven pri-miRNAs including pri-miR-26a-1 using visceral adipose tissue (VAT) from NASH compared to non-NASH cases. These findings indicate that specific miRNAs participate in the pathogenesis of NASH [14]. These studies have thus raised the attention to exploring the significance of miRNAs in NAFLD.

miR-26 is a functional family composed of miR-26a-1, miR-26a-2, and miR-26b subtypes. The expression of miR-26a varied in different kinds of human tumors and showed alteration during developmental and normal tissue growth [15]. Importantly, miR-26a exhibits a dual role in a different kind of cancer, being a tumor suppressor in some [16, 17] and a tumor promoter in others [18]. Furthermore, miR-26a has been reported to regulate pancreatic cell differentiation [19], hepatocyte proliferation during liver regeneration [20], and pathological and physiological angiogenesis [21] in addition to many other vital processes such as autophagy. Formerly, miR-26a was found to participate in modulating immunological functions in mouse models [22, 23]. Previous studies have also indicated that miR-26a played a marked role in regulating the metabolism of glucose, lipids, and insulin sensitivity [24] and pointed to its regulatory effect on oxidative stress caused by hydrogen peroxide produced in vascular smooth muscle cells [25].

Interestingly, one of the genes, which are regulated by miR-26a, is protein kinase delta (PKC*δ*). PKC*δ* is one of the novel protein kinases (*δ*, *ε*, and *θ*) which are activated by diacylglycerol (DAG), a free fatty acid metabolite [26]. Indeed, several studies have shown that a high-fat diet and lipid treatment promoting hepatic triglyceride and DAG accumulation activate PKC*δ* [27]. Likewise, Greene et al. [28] have reported a reduction in hepatic TG accumulation and alteration in hepatic lipogenic gene expression in PKC*δ* null mice. Attenuated oxidative stress and apoptosis were also demonstrated. Additionally, PKC*δ* was previously found to induce ER stress through TNF propagation, which is mediated by JNK activation and induction of CHOP/GADD53 [29]. Moreover, NADPH oxidase complex (p47phox, p67phox, p22phox, and Nox2), one of the main sources of ROS, induced liver injury in response to a high-fat diet [30]. It has been documented that PKC*δ* is involved in the activation (phosphorylation) of most of the components of NADPH oxidase complex [31].

Accordingly, the present study aimed at investigating the potential regulatory role of miR-26a in attenuating the development of free fatty acid- (FFA-) induced hepatic steatosis and hepatocyte injury *in vitro* model of NAFLD. To achieve this goal, we evaluated the effect of miR-26a on triglyceride (TG), cholesterol (CL) deposit accumulations, gene expression of lipid homeostasis, and autophagy marker genes. Moreover, we tested its protective effect against ROS, lipid peroxidation, and apoptosis.

## 2. Materials and Methods

### 2.1. Cell Culture and Transduction of HepG2 Cells

HepG2 cell line was cultured and kept up in tissue culture flask in Roswell Park Memorial Institute (RPMI) 1640 medium supplemented with 10% fetal bovine serum (FBS). Lentiviral hsa-miR-26a or scrambled control miR was manufactured by Applied Biological Materials (Richmond, BC, Canada), to overexpress miR-26a and to establish stable cell lines. The lentiviruses were transduced into HepG2 cells following the manufacturer's instruction. After 2 weeks of puromycin antibiotic (2ug/ml) selection, transduction results were validated by quantitative real-time PCR (qRT-PCR).

### 2.2. Transient Transfection

Cells were seeded in a 6-well plate and incubated overnight at 37°C with 5% CO_2_. miR-26a inhibitor and control miR were synthesized by Applied Biological Materials (Richmond, BC, Canada); the oligonucleotides were transfected into HepG2 cells using Fugene 6 transfection reagent (Promega, USA), according to the manufacturer's instructions. After 24 h of incubation, the medium was removed and fatty acid treatment was performed.

### 2.3. Cell Treatment and FFA Overload

Fat overloading of cells followed previous protocol illustrated by Gómez-Lechón et al. [32]; HepG2 stable cell line at nearly 75% confluency was exposed to a long-chain mixture of FFAs (palmitic acid and oleic acid in ratio 1 : 2) at different concentrations for 24 h. Stock solutions of 10 mM palmitate and 50 mM oleate were prepared in culture medium containing 1% bovine serum albumin (BSA) and were conveniently diluted in culture medium without FBS to obtain the desired final concentrations. The FFA and vehicles were added to HepG2 cells 24 h after seeding.

### 2.4. Oil Red O Staining and Neutral Lipid Quantification

The medium was removed, and cells were washed twice with phosphate-buffered saline (PBS). They were then incubated with 10% formalin for 30 min. Next to fixation, cells were washed twice with double distilled water before adding freshly prepared working Oil red O stain (3 parts of stock Oil red O and 2 parts of water, filtered). 15 min later, the stains were removed and the cells were washed several times until the background stains were unnoticeable. The Oil red O stain was then extracted from cells using 100% isopropanol, and the remaining stain solution was transferred into a 96-well plate to measure the absorbance at 492 nm. Afterward, the cells were washed with PBS and stained with 4′,6-diamidino-2-phenylindole (DAPI) for 15 min. The DAPI-stained cells were evaluated using cytation 3 instrument, and the mean DAPI value for each well was determined. The neutral lipid staining per well was calculated by dividing the absorbance of the Oil red O stain by the mean DAPI.

### 2.5. Measurements of Triglyceride (TG) and Total Cholesterol (CL)

Cellular TG and CL were evaluated using an Infinity TG quantification kit (Thermo Fisher Scientific, USA) and a CL quantification kit (Abcam, USA), according to the manufacturer's instructions. Standard curves were generated, and values obtained were normalized with total protein (ng).

### 2.6. Quantification of Malondialdehyde (MDA) and Total Antioxidant Capacity (TAC)

Lipid peroxidation levels, represented as malondialdehyde (MDA), and cellular total antioxidant capacity (TAC) were quantified using MDA and TAC quantification kits (Abcam, USA), according to the manufacturer's instructions. Concentrations of MDA and TAC were calculated from the standard curves, and values were normalized with total protein (ng).

### 2.7. Determination of Apoptosis by Flow Cytometry

Apoptotic cell death was determined using an Annexin V Fluos staining kit (Abcam, USA) according to the manufacturer's instructions. Samples were analyzed using a BD Accuri C6 Flow Cytometer (BD Bioscience, Bedford, MA, USA).

### 2.8. Quantitative Real-Time Reverse Transcriptase Polymerase Chain Reaction (qRT-PCR)

Total RNA was extracted from the HepG2 cells using an All Prep DNA/RNA/Protein (Qiagen, USA) kit. miR-26a expression level was detected using TaqMan reverse transcription cDNA synthesis kit and TaqMan hsa-miR-26a-5p assay (Thermo Fisher Scientific, USA) according to the manufacturer's instructions. The mRNA was reversely transcribed using a high capacity cDNA reverse transcription kit (Thermo Fisher Scientific, USA), and their expression was examined using SYBR green (BioRad, USA). qRT-PCR was performed using a ViiA7 instrument (Life Technologies, USA), and relative expressions of miR-26a and mRNA were calculated with normalization to U6 snRNA or GAPDH values, respectively, by using the 2DDCt method. Sequences of primers are described in Table 1.

### 2.9. Western Blot

The protein level was quantified by Western blot; cells were washed in PBS and lysed with Radioimmunoprecipitation assay (RIPA) lysis buffer containing a protease inhibitor. Proteins were quantified by a Bicinchoninic Acid method (BCA) Pierce protein assay kit (Thermo Fisher Scientific, USA). Proteins were resolved by SDS-PAGE and blotted onto PVDF membranes. The membranes were blocked with 5% nonfat dry milk and probed with primary antibodies anti-beta-actin 1:5000 (Abcam, Cambridge, USA) or anti-PKC*δ* 1:1000 (Cell Signaling Technology, USA) overnight. The following day, the membranes were incubated with appropriate horseradish peroxidase- (HRP-) conjugated secondary antibodies anti-mouse 1:10000 or anti-rabbit 1:25000, respectively, for 1 h (Abcam, Cambridge, USA). The signals were visualized with an ECL kit (Pierce, Thermo Fisher Scientific, USA) using an X-ray film.

### 2.10. Statistical Analysis

The statistical significance was carried out using GraphPad Prism program version 7.02 (GraphPad Software, USA). Data were expressed as the mean ± SD for at least three separate experiments. Student's *t*-test and one way analysis of variance (ANOVA) were used to compare the differences between two or among more than two groups. Differences were considered statistically significant at ^∗^*P* < 0.05.

## 3. Results

### 3.1. Effect of FFA Treatment on miR-26a Expression Level

**Figure 1:**
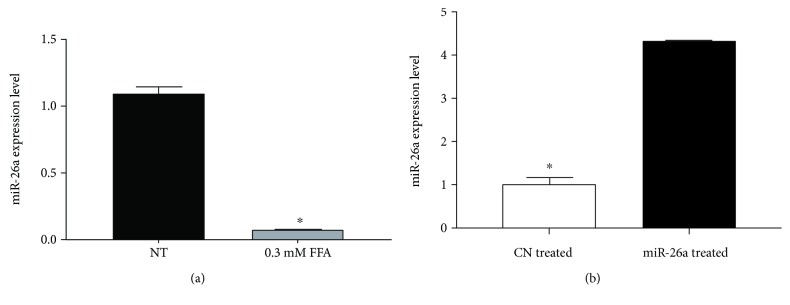
Expression level of miR-26a after (a) FFA treatment of HepG2 cells. HepG2 cells were treated with 0.3 mM PA + OA in ratio 1 : 2 for 24 h. (b) After miR-26a overexpression using lentivirus, U6 snRNA was used as an internal control for miR-26a. Data were expressed as the mean ± SD from three separate experiments (^∗^*P* < 0.05). miR-26a: microRNA-26a; FFA: free fatty acid (PA: palmitic acid + OA: oleic acid); NT: nontreated HepG2 cells; CN treated: scrambled miR transduced stable cells treated with FFA; miR-26a treated: miR-26a transduced stable cells treated with FFA; SD: standard deviation.

miR-26a endogenous expression level significantly decreased upon treatment with FFA when compared to control cells with normal medium (*P* < 0.01, Figure 1(a)). After modifying miR-26a expression levels in HepG2 cells and establishment of stable cell line, miR-26a expression level was markedly increased as compared to scrambled miR control (*P* < 0.01, Figure 1(b)).

### 3.2. Establishment of Steatotic Nonalcoholic Model Using HepG2 Stable Cell Line

**Figure 2:**
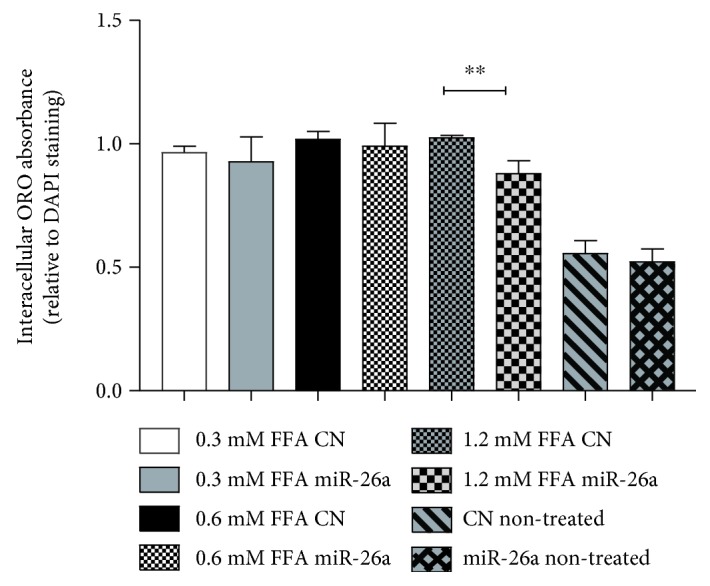
Intracellular Oil red O extract absorbance normalized with mean DAPI measurement in miR-26a stable cell line compared to control stable cell line treated with different FFA concentrations (0.3 mM, 0.6 mM, or 1.2 mM). Data were expressed as the mean ± SD from three separate experiments (^∗∗^*P* < 0.01).

The NAFLD cell model was successfully established by treating HepG2 stable cell line with 1.2 mM FFA for 24 h compared to different concentrations of FFA. We found that 1.2 mM FFA concentration can significantly enhance the neutral lipid accumulation in control cells, and it was significantly downregulated in miR-26a overexpressed cells using Oil red O staining normalized to DAPI staining (*P* < 0.01, Figure 2).

### 3.3. miR-26a Suppresses Triglyceride (TG) and Total Cholesterol (CL) Levels in FFA-Treated Cell

**Figure 3:**
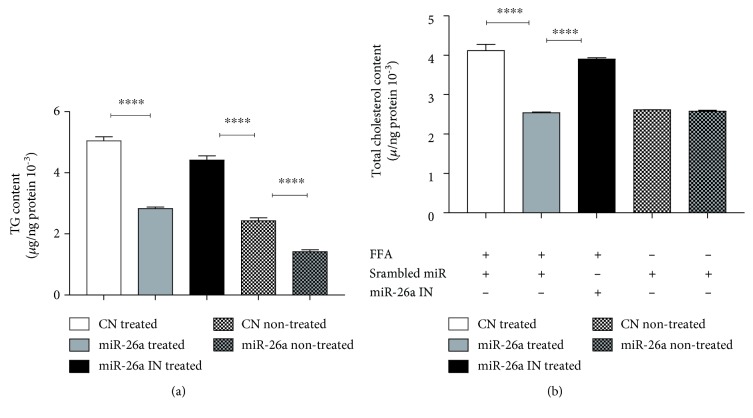
Effect of miR-26a overexpression in stable HepG2 cells on (a) total triglycerides and (b) total cholesterol. CN treated: scrambled miR transduced stable cells treated with FFA and transient transfection of control miR; miR-26a treated: miR-26a transduced stable cells treated with FFA and transient transfection of control miR; miR-26a IN treated: miR-26a transduced stable cells treated with FFA and transient transfection of miR-26a inhibitor; CN nontreated: scrambled miR transduced stable cells with transient transfection of control miR but without FFA treatment; miR-26a nontreated: miR-26a transduced stable cells with transient transfection of control miR but without FFA treatment. Data were collected after 24 h treatment. Results shown are the mean ± SD (^∗∗∗∗^*P* < 0.0001).

We elucidated the regulatory effect of miR-26a on TG accumulation and CL level after treatment with FFA for 24 h. The results showed significantly lower TG accumulation in both conditions: FFA-treated and nontreated cells along with a significant reduction in CL level in FFA-treated cells after miR-26a overexpression, compared with miR control. As expected, the inhibition of miR-26a significantly reversed the effect (Figures 3(a) and 3(b)).

### 3.4. Effect of miR-26a on Lipid Metabolism in FFA-Treated Cell

**Figure 4:**
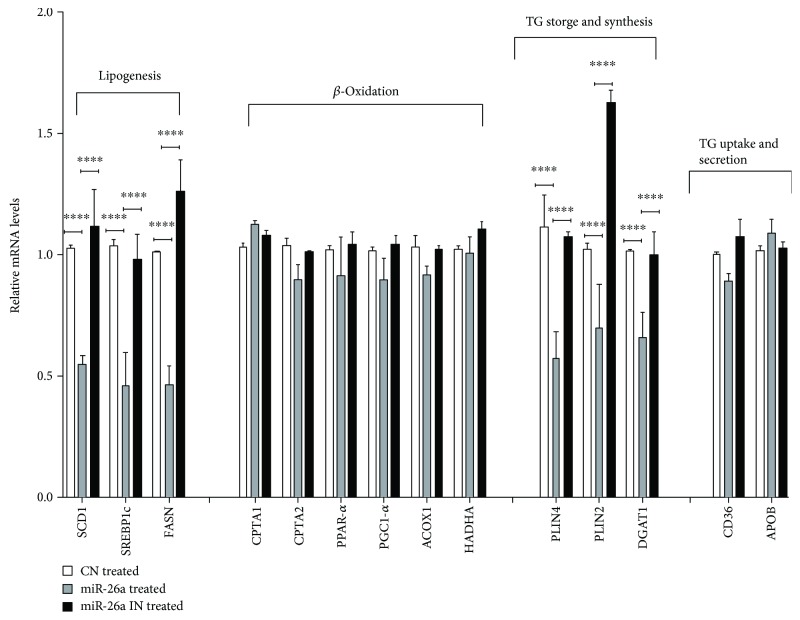
Effect of miR-26a overexpression on expression of genes involved in lipogenesis. FASN, SCD1, and SREBP1c; *β*-oxidation CPTA1, CPT2, PPAR-*α*, PGC1-*α*, ACOX1, and HADHA; triglyceride synthesis and storage PLIN2, PLIN4, and DGAT1; and expression of genes involved in fatty acid uptake transporters as well as TG excretion key marker genes CD36 and ApoB. Group labels are the same as for Figure 3. Results shown are the mean ± SD (^∗∗∗^*P* < 0.001 and ^∗∗∗∗^*P* < 0.0001).

To investigate the modulatory effect of miR-26a on lipid metabolism, lipid metabolism-associated genes were assessed and a comparison between the miR control, miR-26a overexpression, and miR-26a inhibition group was carried out. Gene expression levels of *FASN*, *SCD1*, *SREBP1c*, *DGAT*, *PLIN4* (*P* < 0.0001), and *PLIN2* (*P* < 0.001) were dramatically reduced after miR-26a overexpression in HepG2 cells (Figure 4). Conversely, inhibition of miR-26a in HepG2 cells increased their mRNA expression levels (*P* < 0.0001, Figure 4). Contrarily, genes involved in *β*-oxidation and TG excretions showed insignificant changes after FFA treatment (Figure 4).

### 3.5. miR-26a Reduces PKC*δ* Expression at Both mRNA and Protein Levels

**Figure 5:**
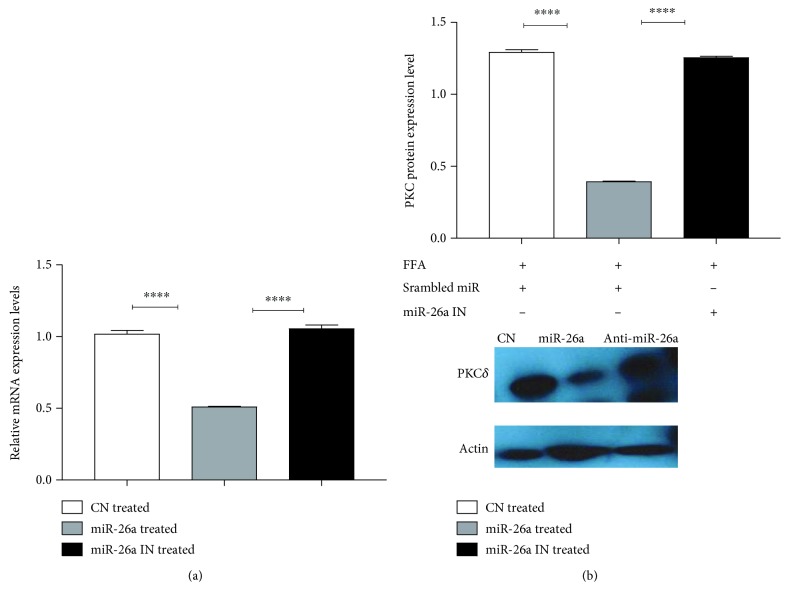
Effect of miR-26a overexpression on (a) PKC*δ* mRNA expression and (b) PKC*δ* protein level. Group labels are the same as for Figure 3. Results shown are the mean ± SD (^∗∗∗∗^*P* < 0.0001).

Figures 5(a) and 5(b) show that endogenous PKC*δ* expression was repressed by miR-26a at mRNA and protein levels when compared to miR control (*P* < 0.0001). Furthermore, the inhibition of miR-26a showed reversing of this downregulation (*P* < 0.0001).

### 3.6. miR-26a Overexpression Protects against Oxidative Stress and Apoptosis in FFA-Treated Cells

**Figure 6:**
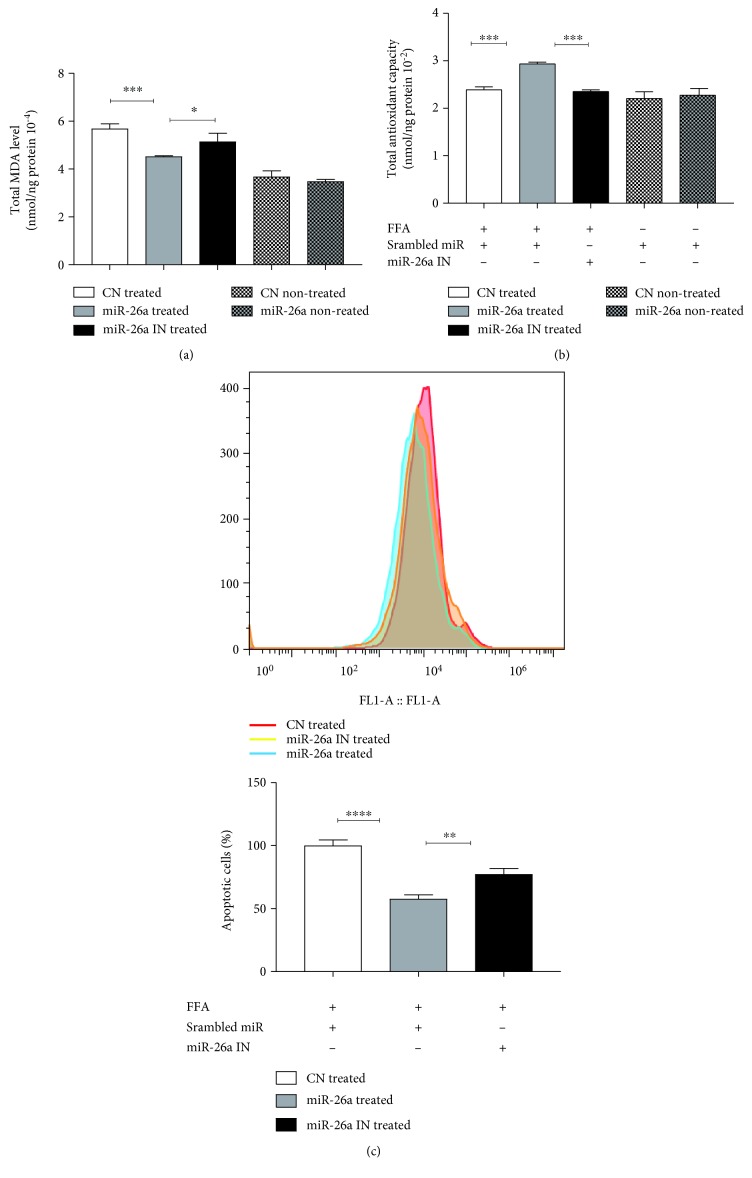
Effect of miR-26a overexpression on (a) total MDA level, (b) total antioxidant level, and (c) apoptosis level indicated by flow cytometry. Group labels are the same as for Figure 3. Results shown are the mean ± SD (^∗^*P* < 0.05, ^∗∗^*P* < 0.01, ^∗∗∗^*P* < 0.001, and ^∗∗∗∗^*P* < 0.0001).

Hepatic levels of lipid peroxidation in terms of MDA were examined. The data in Figure 6(a) showed that miR-26a overexpression decreased the levels of hepatic MDA after FFA treatment when compared to miR control, while miR-26a inhibition totally reverses this result (*P* < 0.001). Moreover, TAC levels were dramatically increased in cells with miR-26a overexpression as compared to miR control. Again, this effect was reversed after transfection with anti-miR-26a (*P* < 0.001). Cell apoptosis was also investigated using flow cytometry. As demonstrated in Figure 6(c), there was a marked reduction of apoptotic cells following miR-26a overexpression when compared to miR control (*P* < 0.0001). Similarly, miR-26a inhibition reversed the effect (*P* < 0.01).

### 3.7. Effect of miR-26a Overexpression on ER Stress, Proinflammatory, Fibrogenic, and Autophagic Markers in FFA-Treated Cells

**Figure 7:**
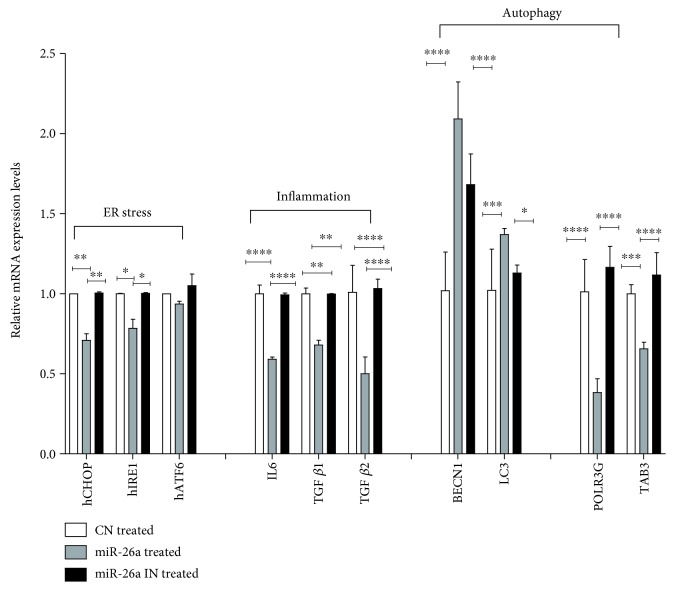
Effect of miR-26a overexpression on expression of genes involved in ER stress markers hCHOP, hIRE1, and hATF6; expression of genes involved in inflammatory marker and IL-6 and fibrosis markers TGF*β*1 and TGF*β*2; mRNA expression of genes involved in autophagy BECN1 and LC3-II; and mRNA expression of POLR3G and TAB3. Group labels are the same as for Figure 3. Results shown are the mean ± SD (^∗^*P* < 0.05, ^∗∗^*P* < 0.01, ^∗∗∗^*P* < 0.001, and ^∗∗∗∗^*P* < 0.0001).

Hepatic expression of several endoplasmic reticulum stress (ER) markers, proinflammatory, fibrogenic mediators, and autophagic markers is examined and demonstrated in Figure 7. Obviously, miR-26a overexpression significantly decreased mRNA expression levels of ER stress markers, namely, *CHOP* (*P* < 0.01) and *IRE1* (*P* < 0.05); the levels of the proinflammatory marker; *IL-6* (*P* < 0.0001) and fibrogenic markers; and *TGFβ1* (*P* < 0.01) and *TGFβ2* (*P* < 0.0001) after 24 h of FFA treatment relative to miR-negative control. However, these modulations in mRNA expression levels were reversed after transfection with miR-26a inhibitor (Figure 7).

The effect of miR-26a overexpression on autophagic markers was also investigated. As shown in Figure 7, miR-26a overexpression upregulated *BECN1* (*P* < 0.0001) and *LC3* (*P* < 0.001) and significantly downregulated the mRNA expression levels of autophagy-negative regulatory genes, *TAB3* (*P* < 0.001) and *POLR3G* (*P* < 0.0001), when compared to miR control. miR-26a inhibition totally reversed the changes in mRNA expression levels (Figure 7).

## 4. Discussion

In this study, we evaluated the regulatory effect of miR-26a on lipid metabolism using the *in vitro* NAFLD model. miR-26a overexpression significantly decreased the levels of TG, CL, and MDA in FFA-treated HepG2 cells, whereas it caused a significant increase in TAC relative to the control. These effects were associated with a significant reduction in the expression of lipogenesis, TG synthesis, storage, and autophagy marker genes. In addition, we observed diminutions in apoptosis level after miR-26a overexpression. All these findings were reversed after treatment with antimiR-26a to confirm the role of miR-26a in the NAFLD model.

Herein, a downregulation of miR-26a was observed in HepG2 cells treated with FFA as an *in vitro* model of NAFLD. Our results were consistent with the previous study [24], which demonstrated decreased hepatic miR-26a in a DIO mouse model after 16 weeks on a high-fat diet (HFD) compared to control mice fed with a standard chow diet (CD). On the other hand, the current data revealed that overexpression of miR-26a attenuated TG accumulation and CL level, in parallel with the decreases in mRNA levels of genes involved in lipogenesis (*SCD1*, *FASN*, and *SREBP1c*), TG synthesis (*DGAT1*), storage (*PLIN2* and *PLIN4*), and insulin signaling (*PKCδ*).

It is worthily noted that NAFLD usually starts with fat deposition in hepatocytes due to metabolic alterations including increased de novo lipogenesis [33]. Previous studies have suggested that *DGAT1* and *PLIN2* mRNA levels increased in livers of humans and rodents with NAFLD [34, 35]. Villanueva et al. [36] have also indicated that *DGAT1* null mice had reduced levels of *SREBP1c* and other lipogenesis enzymes. Another study conducted by Libby et al. [37] showed that loss of *PLIN2* in PLIN2-null mice model prevents diet-induced steatosis upon feeding on a Western diet for 30 weeks, an effect that seemed to be mediated via alteration in the *SREBP-1* and *SERBP-2* pathways. Additionally, it has been reported that miR-26a regulates genes involved in fatty acid, cholesterol metabolism and insulin signaling such as *ACSL3*, *ACSL4*, *PKCδ*, *PKCθ*, *GSK3β*, and *SERBF1*, showing its crucial role in preventing the development of type 2 diabetes mellitus, as some of those genes are direct targets for miR-26a and others are downstream of its target genes [24].

Several research studies in human and animals pointed to the recruitment of oxidative stress biomarkers and lipid peroxidation products in NAFLD [38–40]. NAFLD is usually associated with mitochondrial abnormalities that trigger ROS formation [41, 42]. ROS along with excessive lipid accumulation induce oxidative stress and initiate endoplasmic reticulum (ER) stress [43]. MacDonald et al. [44] showed the ROS could initiate lipid peroxidation and stellate cell activation, leading to inflammation and fibrosis. In tune, this study showed the miR-26a overexpression significantly decreased MDA level and increased TAC after HepG2 cell treatment with FFA. This effect of miR-26a on lipid peroxidation was previously addressed [45].

Likewise, previous *in vitro* and *in vivo* studies using the NAFLD animal model showed an intensification of ER stress and induction of apoptosis [46, 47]. The accumulation of the unfolded proteins sensitizes ER transmembrane signaling proteins to start their response. These proteins include the activating transcription factor 6 (*ATF6*), inositol-requiring enzyme (*IRE-1α*), and PKR-like ER kinase (*PERK*) as well as the proapoptotic transcription factor or C/EBP homologous, which is activated by *IRE-1α* [48, 49].

Interestingly, in line with previous studies [50, 51], miR-26a overexpression decreased the apoptotic fraction and the gene expression of ER stress key marker protein *IRE-1α* and the proapoptotic protein *CHOP*. Meanwhile, all these markers were reversed after miR-26a inhibition.

The preventive role of miR-26a could be related to the regulatory effect of PKC*δ*. The current study confirmed the previously reported role of miR-26a in downregulating PKC*δ* [24, 52].

Moreover, our results determined that miR-26a overexpression downregulated the mRNA expression levels of inflammatory marker *IL-6* and fibrogenic markers *TGFβ1* and *TGFβ2.* In an agreement, former studies have shown that miR-26a inhibited the production of some inflammatory mediators such as *IL-6* and *IL-17*, while it attenuated fibrogenesis under both *in vitro* and *in vivo* conditions [45, 53].

Furthermore, it has been demonstrated that there was an alteration in autophagic flux in livers isolated from both human patients and murine models of NAFLD [54, 55]. The present data showed that miR-26a overexpression induced autophagic flux manifested by upregulation of *BECN1* and *LC3* mRNA expression levels and downregulation of mRNA expression of autophagic inhibitors *TAB3* and *POLR3G*. These findings were consistent with the study described by Han et al. [56] that showed the effect of miR-26a in ameliorating alcoholic hepatic steatosis through induction of autophagy.

In conclusion, the study revealed that miR-26a attenuated triglyceride accumulation through repression of lipogenic genes, TG synthesis, storage, and induction of autophagy. Additionally, miR-26a provides a protective effect against oxidative stress, inflammation, fibrogenesis, and apoptosis induced by FFA loaded in HepG2 cells. Eventually, the study warrants further *in vivo* researches and clinical trials to support the use of miR-26a in the management and prevention of NAFLD.

## Figures and Tables

**(a):** 

Gene	Forward primer (5′-3′)	Reverse primer (5′-3′)
GAPDH	GAAGGTGAAGGTCGGAGTCAA	CAGAGTTAAAAGCAGCCCTGGT
SREBP1c	CAGCCCACTTCATCAAGG	ACTGTTGCCAAGATGGTTCCG
FASN	AACTCCTGCAAGTTCTCCGA	GCTCCAGCCTCGCTCTC
SCD1	GACGATGAGCTCCTGCTGTT	CTCTGCTACACTTGGGAGCC
CD36	GGCTGTGACCGGAACTGTG	AGGTCTCCAACTGGCATTAGAA
DGAT1	TATTGCGGCCAATGTCTTTGC	CACTGGAGTGATAGACTCAACCA
PLIN2	ATGGCATCCGTTGCAGTTGAT	GGACATGAGGTCATACGTGGAG
PLIN4	GGAGCTGCAACCTTCGGAAA	GGACCACTCCCTTAGCCAC
ApoB	AGAGGACAGAGCCTTGGTGGAT	CTGGACAAGGTCATACTCTGCC
IL-6	GTGGAGATTGTTGCCATCAACG	CAGTGGATGCAGGGATGATGTTCT
TGF*β*1	CCCAGCATCTGCAAAGCTC	GTCAATGTACAGCTGCCGCA
TGF*β*2	CAGCACACTCGATATGGACCA	CCTCGGGCTCAGGATAGTCT
hATF6	AGCAGCACCCAAGACTCAAAC	GCATAAGCGTTGGTACTGTCTGA
hCHOP	AGGAACCAGGAAACGGAAACAGA	TCTCCTTCATGCGCTGCTT
hIRE1	GCCGAAGTTCAGATGGAATC	ATCTGCAAAGGCCGATGA
BECN1	GGTGTCTCTCGCAGATTCATC	TCAGTCTTCGGCTGAGGTTCT
LC3	GATGTCCGACTTATTCGAGAGC	TTGAGCTGTAAGCGCCTTCTA
POLR3G	GAGGACGTGCTGCTTATACCT	CTGTTCTGCGGCATCATCGT
TAB3	TGTACTCCATCACCATCTCCT	TGCTTTGCTAACCTCTCCAT
PKC*δ*	AAAGGCAGCTTCGGGAAG	TGGATGTGGTACATCAGGTC

**(b):** 

TaqMan miRNA assay (5′-3′)	
hsa-miR-26a-5p	UUCAAGUAAUCCAGGAUAGGC
U6 snRNA	GTGCTCGCTTCGGCAGCACATATACTAAAATTGGAACGATACAGAGAAGATTAGCAT GGCCCCTGCGCAAGGATGACACGCAAATTCGTGAAGCGTTCCATATTTT

## Data Availability

The data used to support the findings of this study are available from the corresponding author upon request.
